# Supplementary material for: The role of lipid phase and temperature in proton barrier and proton migration on biological membranes
Source: Chem Sci. 2025 Feb 17;16(12):5194–204. doi: 10.1039/d4sc07044e (PMC11841289; doi:10.1039/d4sc07044e)
Supplement: SC-016-D4SC07044E-s001 [file SC-016-D4SC07044E-s001.pdf]

Supplementary Information (SI) for Chemical Science.
This journal is © The Royal Society of Chemistry 2025

Supporting Information

The role of lipid phase and temperature in proton barrier and proton migration on biological membranes

Ambili Ramanthrikkovil Variyam,[1] Mateusz Rzycki,[2] Ramesh Nandi,[1] Alexei A. Stuchebrukhov,[3] Dominik Drabik,[2] and Nadav Amdursky[1,4,*]

[1]Schulich Faculty of Chemistry, Technion – Israel Institute of Technology, Haifa, 3200003, Israel

[2]Department of Biomedical Engineering, Wroclaw University of Science and Technology, Wroclaw, 50-370, Poland

[3]Department of Chemistry, University of California at Davis, One Shields Avenue, Davis, California 95616, USA

[4]School of Mathematical and Physical Sciences, University of Sheffield, Sheffield S3 7HF, United Kingdom

*Corresponding author: Nadav Amdursky

Email: amdursky@technion.ac.il; n.amdursky@sheffield.ac.uk

Table S1: The ground state and excited state pKa of C_{12}-HPTS inside all the lipid mixtures with the absorption and emission maxima for ROH and RO^- states.

Liposome	Absorption (nm)		Emission (nm)		pK_a	ΔpK_a	pK_a^*
	λ_{ROH}	λ_{RO-}	λ_{ROH}	λ_{RO-}			
PC	423	501	471	547	8.13	6.99	1.14
PA:PC 1:9	420	503	471	544	9.34	7.15	2.19
PA:PC 1:3	422	502	474	540	9.65	6.70	2.95
PA:PC 1:1	418	501	474	540	10.13	6.90	3.23
PA:PC 3:1	413	494	465	541	11.35	7.37	3.98
PA	412	486	460	541	11.83	7.33	4.50

Table S2: The ratio of RO^{-*} to ROH* at different temperatures for all the membranes.

Liposome	RO^{-*}/ROH*						
	**10**°C	**20**°C	**30**°C	**40**°C	**50**°C	**60**°C	**70**°C
PC	2.49	3.27	4.93	6.58	8.69	11.36	14.92
PA:PC 1:9	1.34	1.60	2.00	2.59	3.17	3.98	5.00
PA:PC 1:3	1.48	1.81	2.29	2.79	3.34	4.11	5.13
PA:PC 1:1	2.28	2.70	3.25	4.29	5.00	6.10	7.46
PA:PC 3:1	3.58	4.08	4.90	5.71	7.04	9.26	11.11
PA	3.87	4.72	5.35	6.90	12.50	18.18	21.28

Table S3: k_{PT} (extracted from short-time kinetics), B, d, and τ_0 (extracted from long-time kinetics) as a function of temperature for all the mixtures.

**Temperature (°C)**	**Short time kinetics**	**Long time kinetics**		
	k_{PT} (ns^{-1})	B	*d*	τ_0 (ns)
PC				
10	6.2	2.5	2.4	1.8
20	6.5	2.2	2.45	1.5
30	6.7	1.9	2.7	0.9
40	6.8	1.7	2.8	0.7
50	6.9	1.6	2.9	0.5
60	7.0	1.5	3.0	0.4
70	7.1	1.45	3.1	0.3
PC:PA 9:1				
10	5.5	2.8	1.4	1.8
20	5.7	2.7	1.5	1.4
30	6.2	2.35	1.8	1.0
40	6.4	2.2	2.0	0.9
50	6.5	2	2.2	0.8
60	6.6	1.9	2.3	0.7
70	6.7	1.8	2.6	0.5
PC:PA 3:1				
10	5.8	2.7	1.4	1.7
20	5.9	2.6	1.5	1.6
30	6.3	2.5	1.8	1.5
40	6.6	2.3	2.0	1.2
50	6.7	2.2	2.2	1.1
60	6.8	2.1	2.4	0.9
70	6.9	2.0	2.6	0.8
PC:PA 1:1				
10	6.0	2.5	1.5	1.5

20	6.1	2.4	1.6	1.4
30	6.3	2.1	1.7	1.3
40	6.8	1.9	2.0	1.0
50	6.9	1.7	2.2	0.8
60	7.0	1.65	2.3	0.7
70	7.1	1.6	2.5	0.5
PC:PA 1:3				
10	6.7	2.5	1.1	1.4
20	6.8	2.3	1.2	1.3
30	6.9	2.1	1.25	1.25
40	7.0	1.95	1.5	1.1
50	7.7	1.8	1.8	1.0
60	7.9	1.7	2.0	0.9
70	8.0	1.65	2.2	0.8
PA				
10	6.8	1.9	1.4	1.4
20	6.9	1.7	1.5	1.3
30	7.0	1.55	1.6	1.2
40	7.1	1.5	1.7	0.9
50	7.2	1.4	1.75	0.5
60	8.4	1.2	2.0	0.3
70	8.6	1.1	2.1	0.2

Table S4: k_{PT}^{-1} calculated for all the mixtures at different temperatures.

**Liposome**	$\mathbf{K_{PT}^{-1}}$						
	**10**°C	**20**°C	**30**°C	**40**°C	**50**°C	**60**°C	**70**°C
PC	2.27	1.78	1.15	0.82	0.58	0.40	0.26
PA:PC 1:9	3.92	3.39	2.92	2.30	1.88	1.49	1.17
PA:PC 1:3	3.74	3.06	2.56	2.18	1.82	1.47	1.16
PA:PC 1:1	2.44	2.06	1.74	1.39	1.18	0.95	0.76
PA:PC 3:1	1.68	1.48	1.04	1.04	0.91	0.67	0.53
PA	1.55	1.26	1.11	0.83	0.37	0.26	0.20

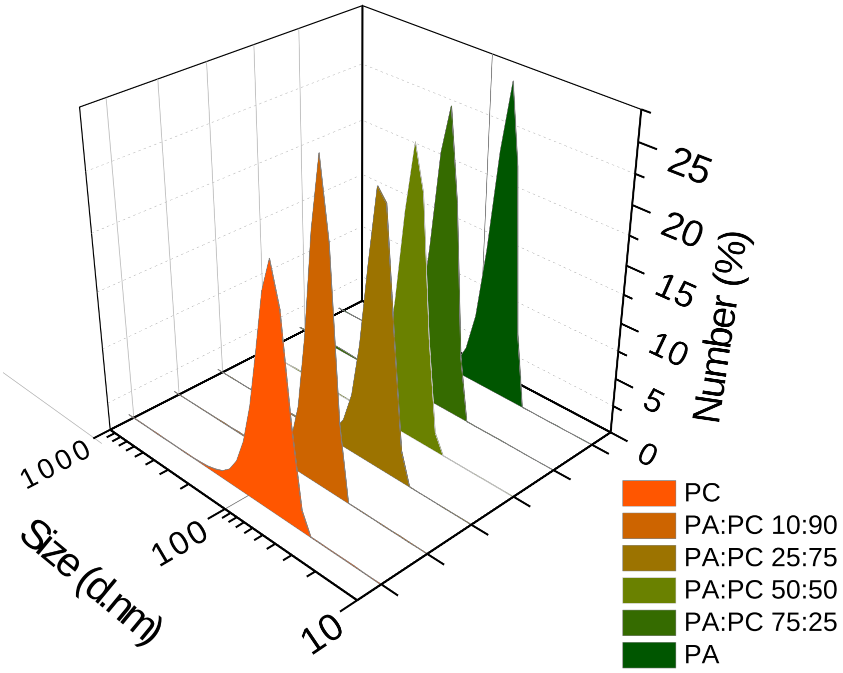

Figure S1: DLS measurements of all the mixtures showing the formation of monodispersed solutions of SUVs after extrusion.

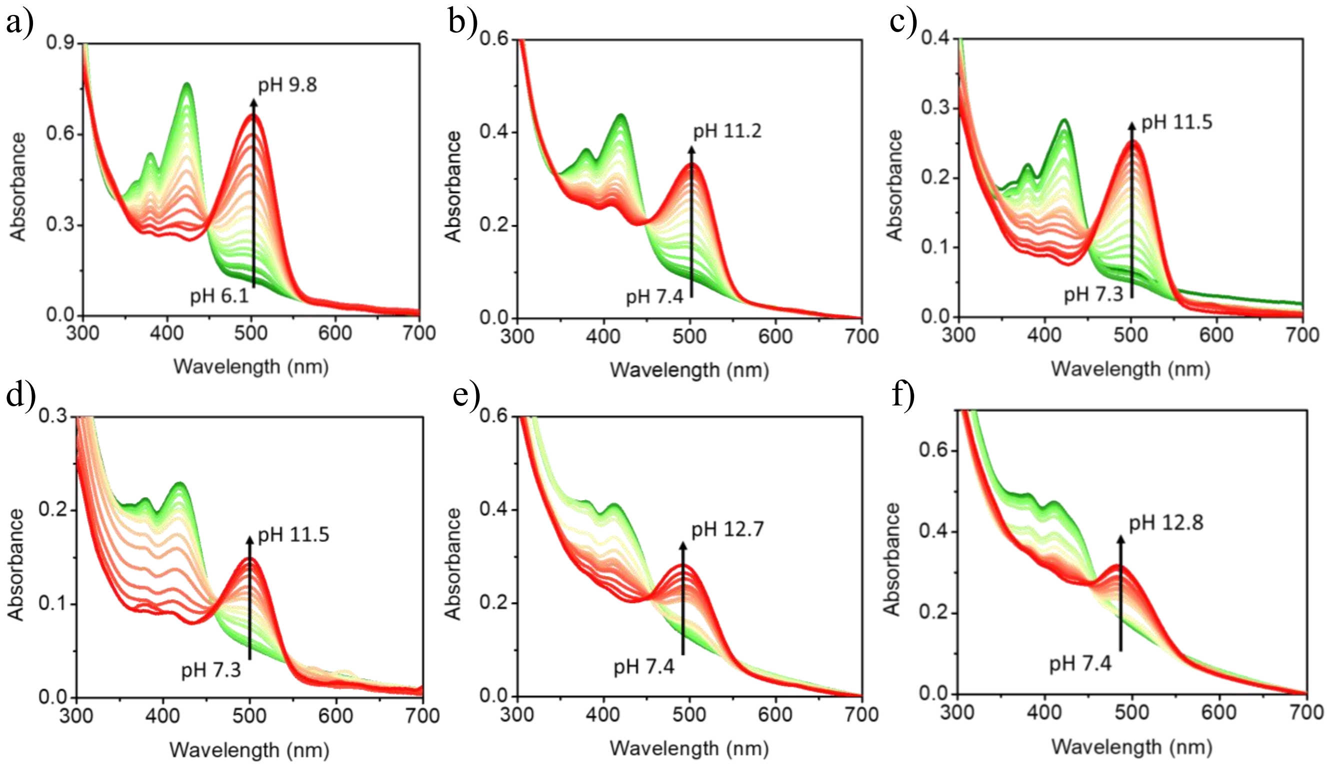

Figure S2: pH titration experiments carried out using UV-visible absorption spectroscopy showing the absorption of probe at different pHs for (a) DMPC, (b) DMPC:DMPA 9:1, (c) DMPC:DMPA 3:1, (d) DMPC:DMPA 1:1, (e) DMPC:DMPA 1:3, and (f) DMPA.

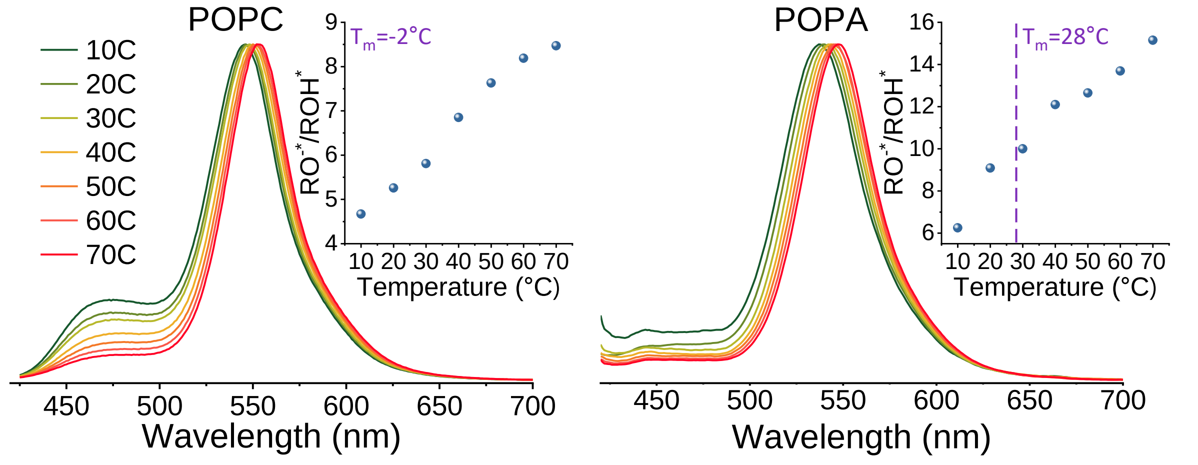

Figure S3: Temperature-dependent **s**teady-state emission spectra of C_{12}-HPTS inside POPC (right) and POPA (left). Insets showing the ratio of RO^{-}*/ROH* calculated from the emission spectra vs temperature.

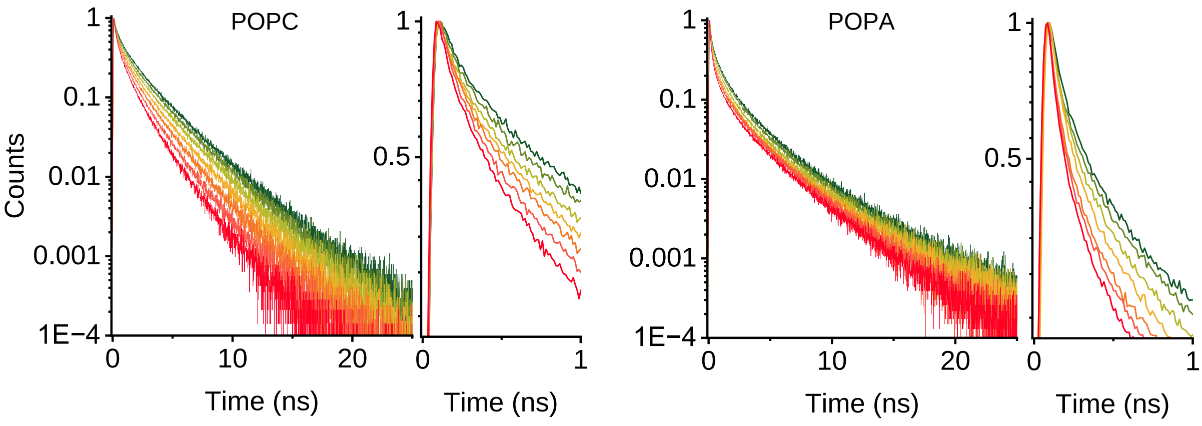

Figure S4: Time-resolved fluorescence showing the temperature-dependent behavior of C_{12}-HPTS inside POPC (right) and POPA (left). Insets showing the fluorescence decays for the first nanosecond.

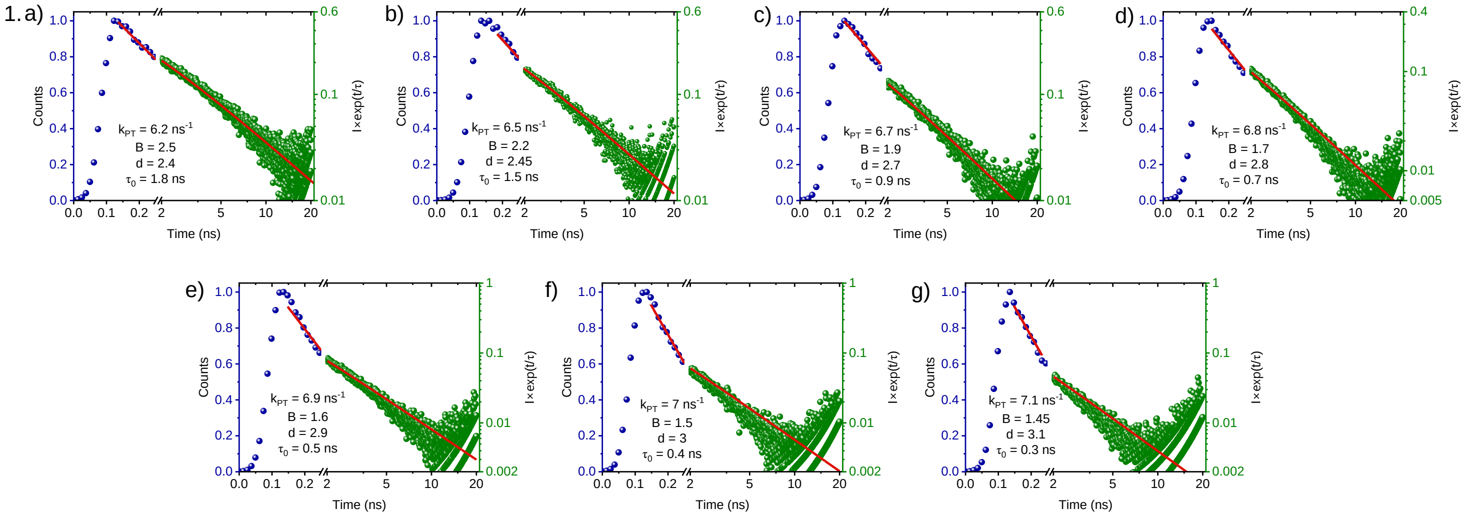
1. a)
k_{PT} = 6.2 ns^{-1}
B = 2.5
d = 2.4
τ_0 = 1.8 ns
b)
k_{PT} = 6.5 ns^{-1}
B = 2.2
d = 2.45
τ_0 = 1.5 ns
c)
k_{PT} = 6.7 ns^{-1}
B = 1.9
d = 2.7
τ_0 = 0.9 ns
d)
k_{PT} = 6.8 ns^{-1}
B = 1.7
d = 2.8
τ_0 = 0.7 ns
e)
k_{PT} = 6.9 ns^{-1}
B = 1.6
d = 2.9
τ_0 = 0.5 ns
f)
k_{PT} = 7 ns^{-1}
B = 1.5
d = 3
τ_0 = 0.4 ns
g)
k_{PT} = 7.1 ns^{-1}
B = 1.45
d = 3.1
τ_0 = 0.3 ns
Counts
I×exp(t/τ)
Time (ns)

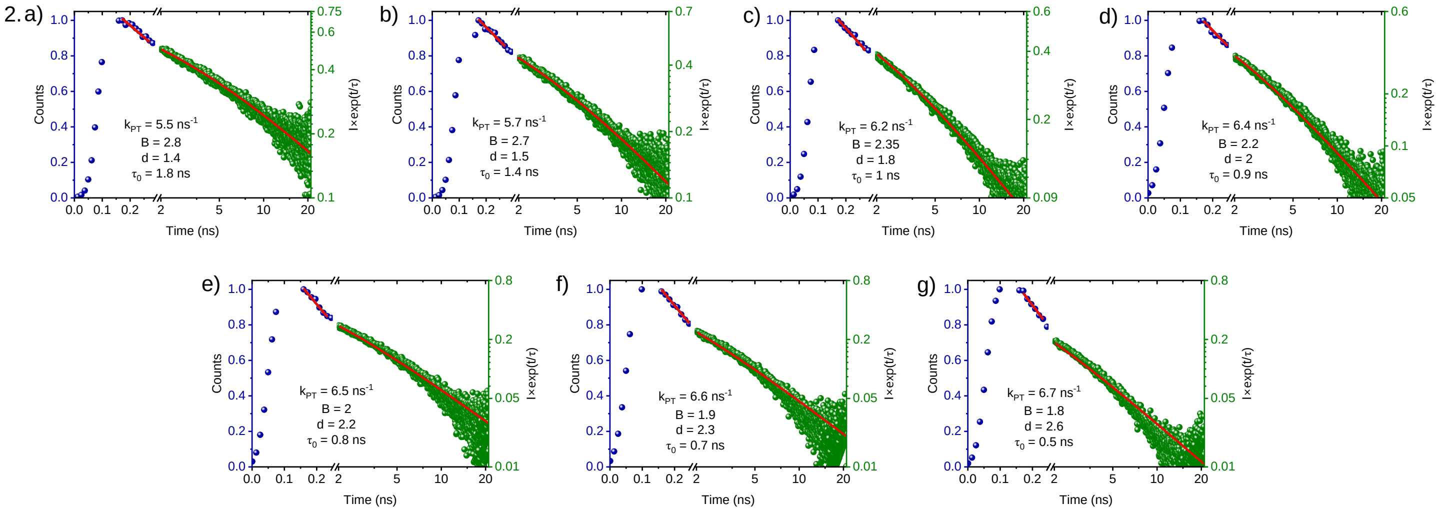
2. a)
k_{PT} = 5.5 ns^{-1}
B = 2.8
d = 1.4
τ_0 = 1.8 ns
b)
k_{PT} = 5.7 ns^{-1}
B = 2.7
d = 1.5
τ_0 = 1.4 ns
c)
k_{PT} = 6.2 ns^{-1}
B = 2.35
d = 1.8
τ_0 = 1 ns
d)
k_{PT} = 6.4 ns^{-1}
B = 2.2
d = 2
τ_0 = 0.9 ns
e)
k_{PT} = 6.5 ns^{-1}
B = 2
d = 2.2
τ_0 = 0.8 ns
f)
k_{PT} = 6.6 ns^{-1}
B = 1.9
d = 2.3
τ_0 = 0.7 ns
g)
k_{PT} = 6.7 ns^{-1}
B = 1.8
d = 2.6
τ_0 = 0.5 ns
Counts
I×exp(t/τ)
Time (ns)

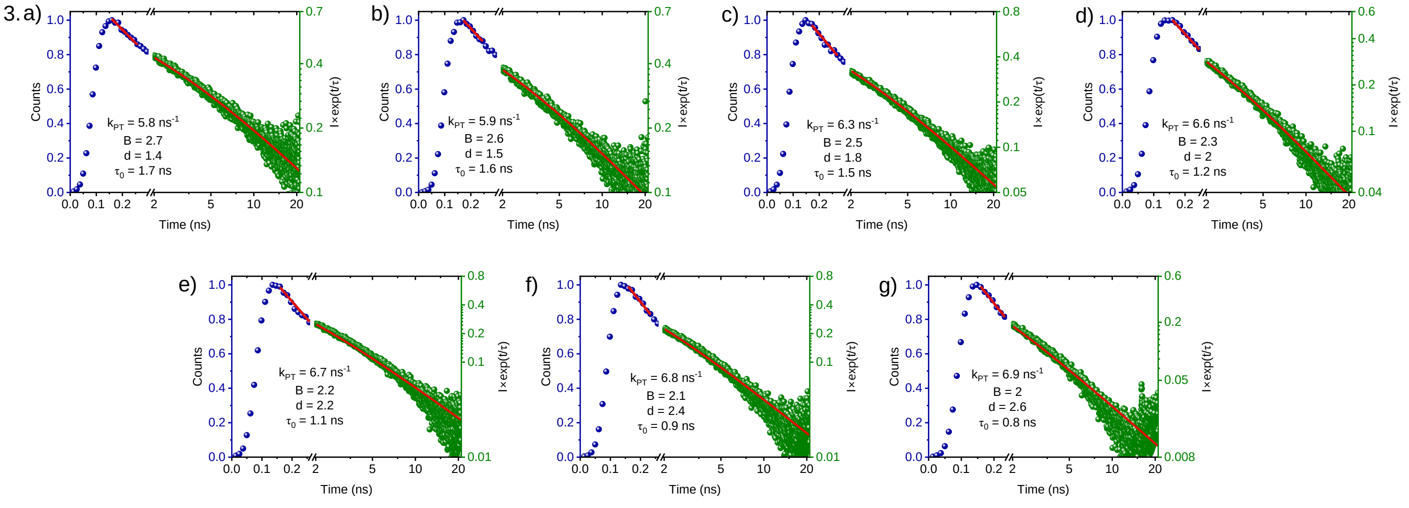
3. a)
k_{PT} = 5.8 ns^{-1}
B = 2.7
d = 1.4
τ_0 = 1.7 ns
b)
k_{PT} = 5.9 ns^{-1}
B = 2.6
d = 1.5
τ_0 = 1.6 ns
c)
k_{PT} = 6.3 ns^{-1}
B = 2.5
d = 1.8
τ_0 = 1.5 ns
d)
k_{PT} = 6.6 ns^{-1}
B = 2.3
d = 2
τ_0 = 1.2 ns
e)
k_{PT} = 6.7 ns^{-1}
B = 2.2
d = 2.2
τ_0 = 1.1 ns
f)
k_{PT} = 6.8 ns^{-1}
B = 2.1
d = 2.4
τ_0 = 0.9 ns
g)
k_{PT} = 6.9 ns^{-1}
B = 2
d = 2.6
τ_0 = 0.8 ns
Counts
I×exp(t/τ)
Time (ns)

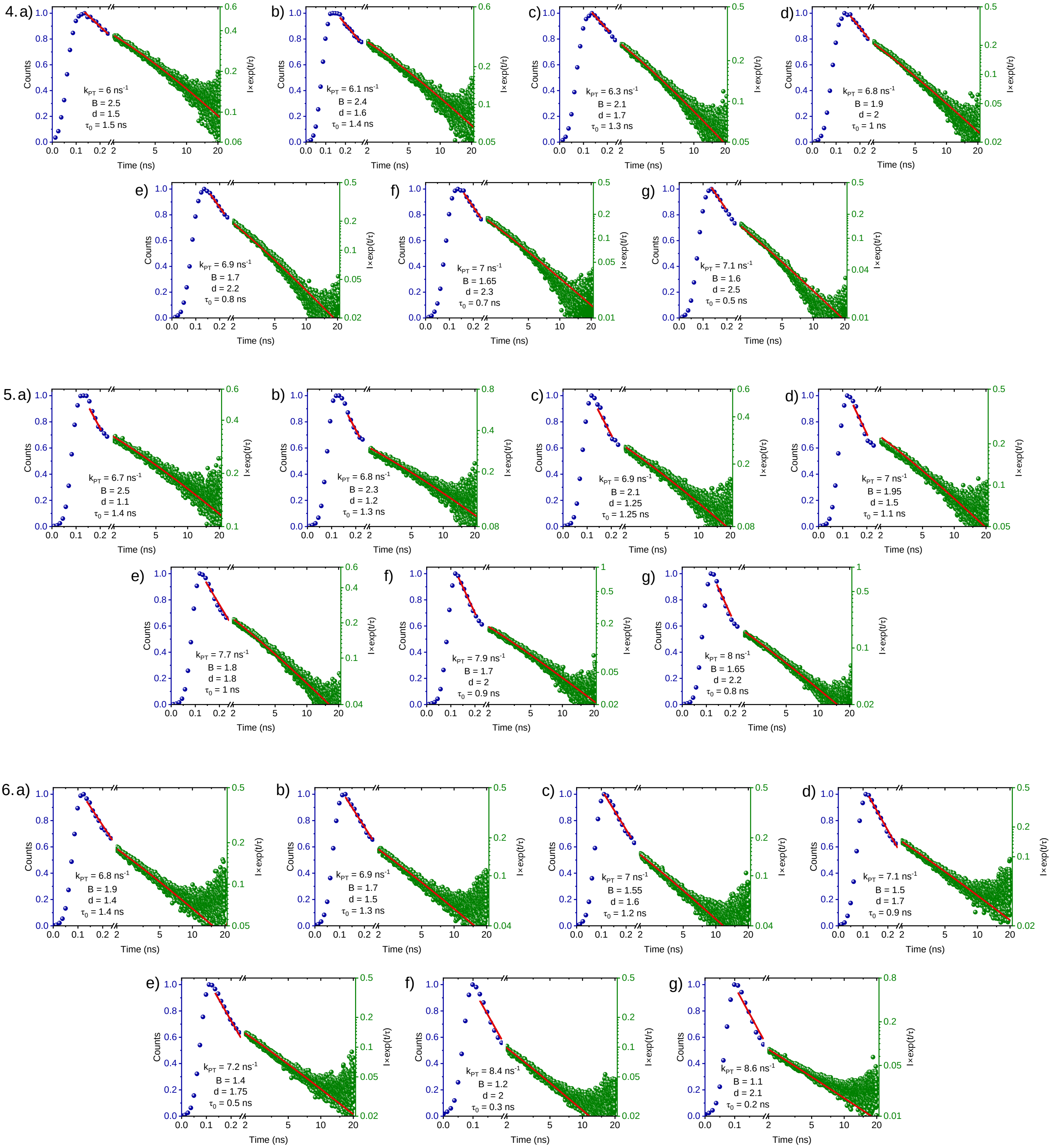

Figure S5: Fitting of all the fluorescence decays of (1) DMPC, (2) DMPC:DMPA 9:1, (3) DMPC:DMPA 3:1, (4) DMPC:DMPA 1:1, (5) DMPC:DMPA 1:3, and (6) DMPA with the theoretical model at (a) 10 ºC, (b) 20 ºC, (c) 30 ºC, (d) 40 ºC, (e) 50 ºC, (f) 60 ºC and (g) 70 ºC. The first few nanoseconds of data are fitted with equation (3) and latter nanoseconds are fitted based on equation (4) as shown in the main text.

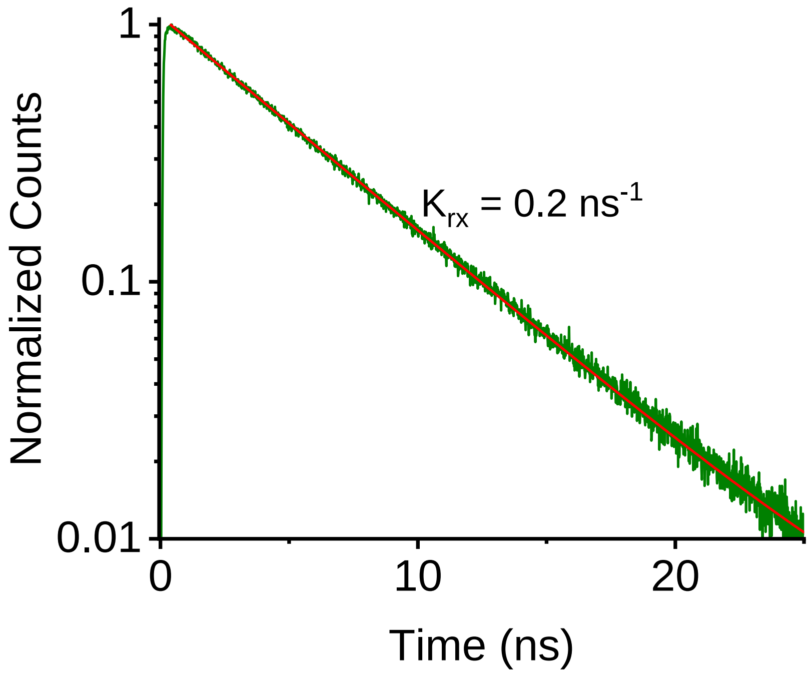

Figure S6: Exponential fitting of the decay of probe inside vesicles at 550 nm to find the rate constant.

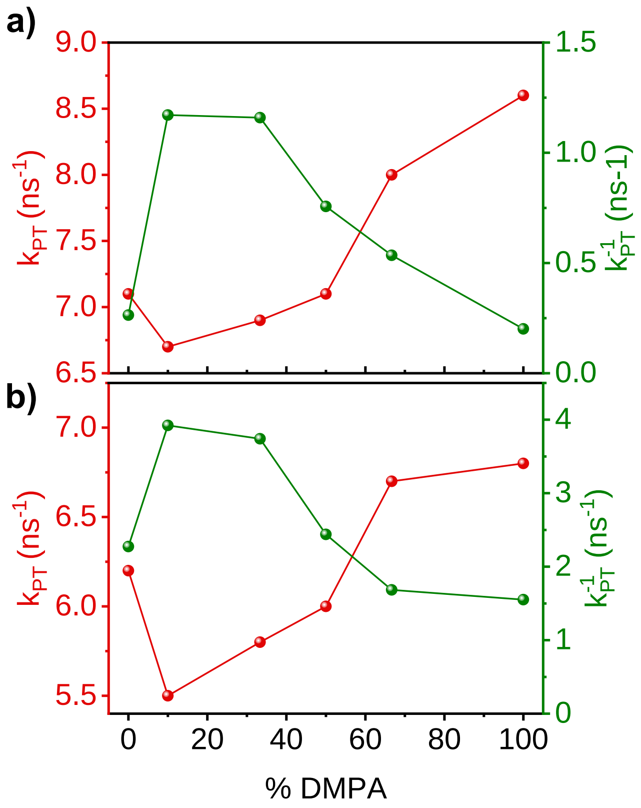

Figure S7: k_{PT} and k_{PT}^{-1} of all the mixture membranes at (a) 70 ºC and (b) 10ºC.

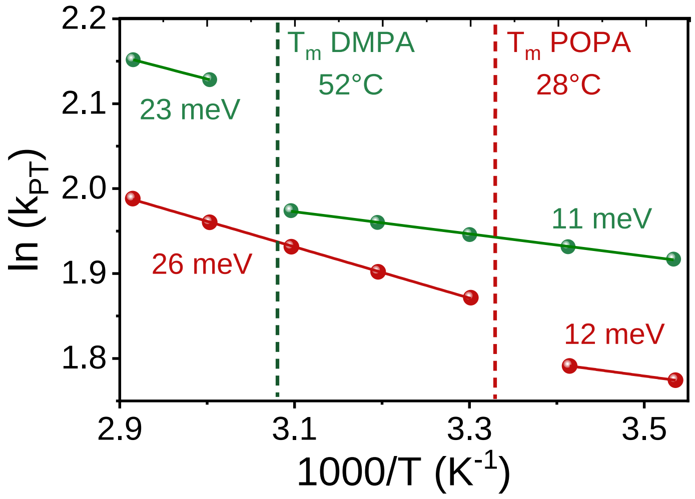

Figure S8: The change of k_{PT} as a function of temperature for POPA (red) in comparison to DMPA (green).

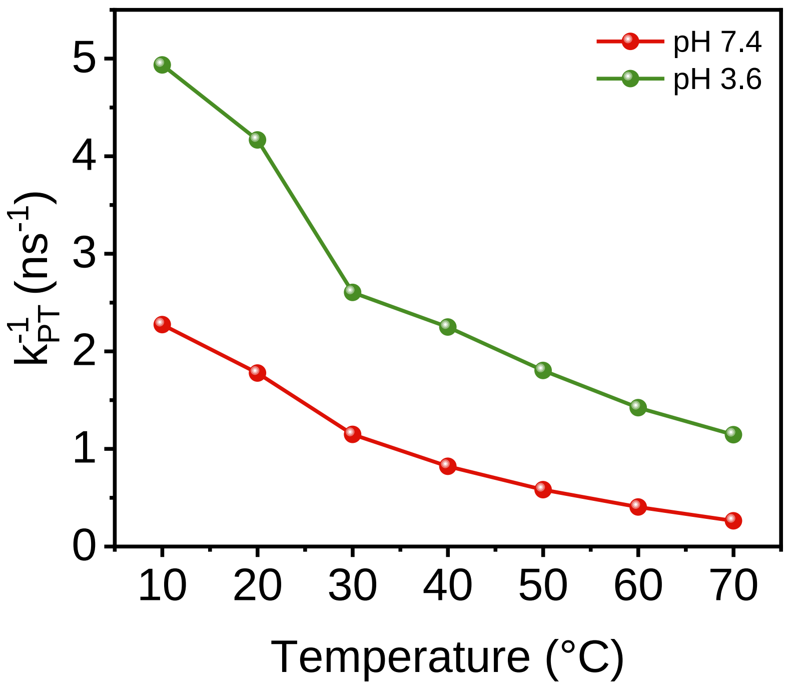

Figure S9: The temperature-dependent change in k_{PT}^{-1} at two different pHs.

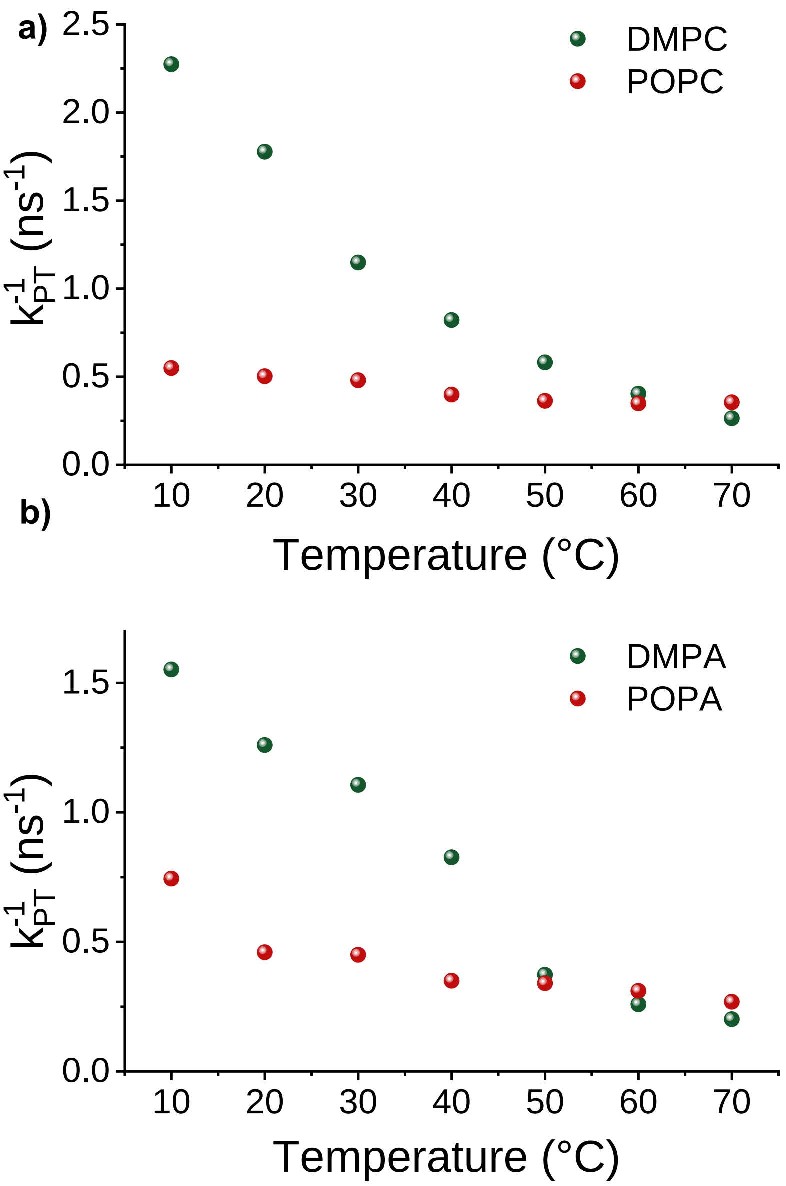

Figure S10. Comparison in the temperature-dependent change of the k_{PT}^{-1} values between (a) DMPC and POPC, and (b) DMPA and POPA.

MD Simulations:

To explore the characteristics and dynamics of DMPA/DMPC bilayers, various membrane systems were generated using the CHARMM-GUI membrane builder.[1] These systems comprised different mixtures of DMPC and DMPA at ratios of 1:0, 0.9:0.1, 0.75:0.25, 0.5:0.5, 0.25:0.75, and 0:1. The lipid bilayers were solvated using the TIP3P water model, maintaining 50 water molecules per lipid to ensure adequate solvation.[2] Each PA/PC system contained 600 lipids, and Na+ ions were added to neutralize the system's total charge. Molecular dynamics

(MD) simulations were performed using the GROMACS 2021 software suite[3] and the CHARMM36 force field.[4]

Initially, energy minimization was performed on the membrane systems using a steepest descent algorithm. Subsequently, an NVT (constant Number of particles, Volume, and Temperature) ensemble was applied, with a leap-frog integrator and a 1 fs time step. In the NPT (constant Number of particles, Pressure, and Temperature) ensemble, a 2-fs time step was used. The simulations employed a Nose-Hoover thermostat across several temperatures: 283, 293, 305, 315, 323, and 343 K, with a time constant of $\tau_t = 2$ ps.[5, 6] Pressure coupling was handled semi-isotropically using the Parrinello-Rahman barostat, set at 1 bar with $\tau_p = 5$ ps.[7] During equilibration, positional and dihedral restraints were applied, with gradually decreasing force constants. Bond lengths were constrained using the LINCS algorithm.[8]

During the production phase, the hydrogen mass repartitioning technique was employed, enabling a 4 fs time step.[9] Van der Waals interactions were smoothly turned off between 1.0 and 1.2 nm through a force-based switching function,[10] while long-range electrostatic interactions were calculated using the particle mesh Ewald (PME) method, with a grid spacing of 0.1 nm and a cutoff distance of 1.2 nm.[11]

Area per Lipid and Membrane Thickness: A custom MATLAB script determined the membrane area per lipid (APL) and membrane thickness (MT). Each lipid molecule was described by the midpoint between P and C2 atoms' position. Such points were used for membrane thickness calculation; specifically, they were calculated as the difference between average height (z-axis) values of described points in opposite leaflets. This was followed by Voronoi tessellation to obtain the individual APL for each lipid molecule in every simulation time step. The APL dataset was histogrammed, and the APL value was obtained from the peak value of the fitted Gaussian function.

Bending Rigidity: The real-space fluctuation (RSF) method was used to determine the bending rigidity of investigated membrane systems.[12] Specifically, for each lipid in each time step a splay was calculated, from which a distribution was calculated. Lipid splay is defined as the divergence of the angle formed by the directors of neighboring lipids providing that they are weakly correlated. The obtained distribution is fitted to obtain the bending rigidity coefficient.

Lateral Diffusion Coefficient: The lateral diffusion of lipid molecules was quantified using the Diffusion Coefficient Tool plugin.[13] This coefficient is derived from Einstein's equation, utilizing the mean square displacement (MSD) of the selected molecular species. Specifically,

the lateral diffusion for each lipid species was computed in the xy-plane, based on the positional data of phosphorus atoms. The analysis followed the procedure outlined in our previous study.[14]

References

1. E. L. Wu, X. Cheng, S. Jo, H. Rui, K. C. Song, E. M. Dávila-Contreras, Y. Qi, J. Lee, V. Monje-Galvan and R. M. Venable, CHARMM-GUI membrane builder toward realistic biological membrane simulations, *J. Comput. Chem.*, 2014, **35**, 1997-2004.
2. W. L. Jorgensen, J. Chandrasekhar, J. D. Madura, R. W. Impey and M. L. Klein, Comparison of simple potential functions for simulating liquid water, *J. Chem. Phys.*, 1983, **79**, 926-935.
3. D. Van Der Spoel, E. Lindahl, B. Hess, G. Groenhof, A. E. Mark and H. J. Berendsen, GROMACS: fast, flexible, and free, *J. Comput. Chem.*, 2005, **26**, 1701-1718.
4. J. B. Klauda, R. M. Venable, J. A. Freites, J. W. O'Connor, D. J. Tobias, C. Mondragon-Ramirez, I. Vorobyov, A. D. MacKerell Jr and R. W. Pastor, Update of the CHARMM all-atom additive force field for lipids: validation on six lipid types, *J. Phys. Chem. B*, 2010, **114**, 7830-7843.
5. W. G. Hoover, Canonical dynamics: Equilibrium phase-space distributions, *Phys. Rev. A*, 1985, **31**, 1695.
6. S. I. NOSÉ, A molecular dynamics method for simulations in the canonical ensemble, *Mol. Phys.*, 2002, **100**, 191-198.
7. M. Parrinello and A. Rahman, Polymorphic transitions in single crystals: A new molecular dynamics method, *J. Appl. Phys.*, 1981, **52**, 7182-7190.
8. B. Hess, H. Bekker, H. J. Berendsen and J. G. Fraaije, LINCS: a linear constraint solver for molecular simulations, *J. Comput. Chem.*, 1997, **18**, 1463-1472.
9. C. W. Hopkins, S. Le Grand, R. C. Walker and A. E. Roitberg, Long-time-step molecular dynamics through hydrogen mass repartitioning, *J. Chem. Theory Comput.*, 2015, **11**, 1864-1874.
10. P. J. Steinbach and B. R. Brooks, New spherical-cutoff methods for long-range forces in macromolecular simulation, *J. Comput. Chem.*, 1994, **15**, 667-683.
11. U. Essmann, L. Perera, M. L. Berkowitz, T. Darden, H. Lee and L. G. Pedersen, A smooth particle mesh Ewald method, *J. Chem. Phys.*, 1995, **103**, 8577-8593.
12. M. Doktorova, D. Harries and G. Khelashvili, Determination of bending rigidity and tilt modulus of lipid membranes from real-space fluctuation analysis of molecular dynamics simulations, *Phys. Chem. Chem. Phys.*, 2017, **19**, 16806-16818.
13. T. Giorgino, Computing diffusion coefficients in macromolecular simulations: the Diffusion Coefficient Tool for VMD, *J. Open Source Softw.*, 2019, **4**, 1698.
14. A. R. Variyam, M. Rzycki, A. Yucknovsky, A. A. Stuchebrukhov, D. Drabik and N. Amdursky, Proton diffusion on the surface of mixed lipid membranes highlights the role of membrane composition, *Biophys. J.*, 2024, 10.1016/j.bpj.2024.1007.1002.
